# Dissecting the pathogenic effects of smoking in blood DNA methylation on allergic diseases^☆^

**DOI:** 10.1016/j.waojou.2024.100995

**Published:** 2024-11-21

**Authors:** Junhao Tu, Wei Wan, Binxiang Tang, Fan Jiang, Jinyang Wen, Qing Luo, Jing Ye

**Affiliations:** a Department of Otorhinolaryngology, Head and Neck Surgery, The First Affiliated Hospital, Jiangxi Medical College, Nanchang University, Nanchang, Jiangxi Province, China; b Department of Otolaryngology, Yong Loo Lin School of Medicine, National University of Singapore, National University Health System, Singapore; c Department of Otorhinolaryngology Head and Neck Surgery, The Second Affiliated Hospital of Nanchang University, Nanchang, Jiangxi Province, China; d Department of Radiology, Tongji Hospital, Tongji Medical College, Huazhong University of Science and Technology, Wuhan, Hubei Province, China; e Department of Allergy, The First Affiliated Hospital, Jiangxi Medical College, Nanchang University, Nanchang, Jiangxi Province, China

**Keywords:** Allergic diseases, Asthma, Smoking, DNA methylation, Mendelian randomization

## Abstract

**Background:**

Allergic diseases, such as asthma and allergic rhinitis, present significant health challenges globally. Elucidating the genetic and epigenetic foundations is crucial for developing effective interventions.

**Methods:**

We performed two-sample Mendelian Randomization (MR) analyses to investigate the associations between smoking behaviors and various allergic diseases, leveraging data from the FinnGen database. Additionally, we examined the relationships of DNA methylation (CpG sites) with allergic diseases, employing mQTLs as epigenetic proxies. Furthermore, we conducted reverse MR analyses on CpG sites that exhibited cross-allergic disease effects.

**Results:**

In our genomic MR analysis, smoking behaviors such as smoking initiation and the number of cigarettes smoked per day were identified to be causally associated with an increased risk of asthma. Additionally, there was suggestive evidence linking smoking initiation to atopic contact dermatitis. Our epigenetic MR analysis found that methylation changes at 46 CpG sites, assessed via mQTLs, were significantly associated with asthma risk. Notably, cg17272563 (PRRT1), cg03689048 (BAT3), cg20069688 (STK19), and cg20513976 (LIME1) were identified with cross-allergic effects. Crucially, reverse MR analysis substantiated these associations.

**Conclusions:**

Our study has highlighted the associations between smoking behaviors and allergic diseases in the genetic and epigenetic landscape, notably asthma. We identified several DNA methylation-related CpG sites, such as cg03689048 (BAT3), cg17272563 (PRRT1), and cg20069688 (STK19), which demonstrate cross-allergic potential and reverse causal relationships.

## Introduction

Allergic diseases are a global health concern,^1^ affecting a substantial portion of the population and manifesting in various forms, such as allergic conjunctivitis (AC), allergic contact dermatitis (ACD), allergic urticaria (AU), allergic purpura (AP), allergic rhinitis (AR), and asthma.^2^^,^^3^ Allergic diseases often progress sequentially and can affect individuals at any stage of life, starting with infantile contact-specific dermatitis, progressing to AR and eventually leading to asthma—the most severe allergic disease.^4^ Smoking is regarded a potential factor that may influence the progression of allergic diseases.^5^, ^6^, ^7^ A previous study reported a higher incidence of asthma among adults with a history of smoking.^8^ However, most randomized controlled trials have excluded smokers with asthma. The impact of smoking on different allergic diseases appears to vary, and there is insufficient evidence to definitively determine the role of smoking in the progression of these diseases.^6^^,^^7^^,^^9^ Notably, in the most observational studies, fully controlling for confounding factors is particularly challenging due to the complex associations between smoking and numerous lifestyle and socioeconomic factors.

Mendelian Randomization (MR) is an innovative epidemiological method that employs common genetic variations as proxies for exposure, allowing for the estimation of causal relationships between modifiable risk factors and health-related traits or diseases.^10^, ^11^, ^12^ A previous MR study explored the causal relationships between metabolites and allergic diseases.^13^ The researchers investigated the correlation between smoking and asthma, but their findings did not provide strong support for their conclusions, while they emphasized the necessity for more definitive research.^14^ Furthermore, the specific biological pathways through which smoking contributes to the pathogenesis of allergic diseases, as well as the consistency of its impact across different allergic diseases, remain unclear. To address these complexities and identify factors that influence allergic diseases, a systematic analysis of common allergic conditions is essential.

Numerous studies have confirmed that smoking can induce changes in the DNA methylation levels across the whole epigenome.^15^, ^16^, ^17^ Concurrently, alterations in DNA methylation are associated with the onset and progression of asthma and other allergic diseases.^18^, ^19^, ^20^, ^21^, ^22^ In the development of allergic diseases development, 1 type of aberrant methylation involves genome-wide hypomethylation.^21^^,^^23^^,^^24^ Hence, it has been suggested that DNA methylation might serve as a responsive epigenetic pathway, linking genetic susceptibility to allergic diseases with smoking.

This study primarily aimed to use two-sample MR analysis to comprehensively elucidate the relationships between the genetic susceptibility to smoking behavior and the risk of asthma and 5 other allergic diseases. Additionally, we investigated the impact of genetically predicted smoking-related DNA methylation on the risk of asthma, as well as 5 other allergic diseases. Subsequently, we conducted genetic colocalization and reverse MR analyses to further elucidate of how smoking exerts its pathogenic effects on allergic diseases, particularly asthma.

## Materials and methods

### Study design

Traditional observational studies exploring the relationships between smoking and allergic diseases are often influenced by confounding factors, such as socioeconomic status and lifestyle, making it difficult to clearly distinguish the causal relationship between smoking and diseases. Although prospective studies can mitigate these issues, they usually require long-term follow-up and large sample sizes, which are difficult to achieve in practice. Additionally, smoking may alter DNA methylation, potentially affecting disease risk—a mechanism that is challenging to isolate and confirm in traditional research. The MR method, by using genetic variants associated with smoking behavior and DNA methylation as instrumental variables (IVs), can minimize the effects of confounding factors and reverse causality, while also revealing the potential causal mechanisms of smoking on DNA methylation and its relationship with allergic diseases. Thus, we chose the MR method not only to more accurately infer the causal relationships between smoking and allergic diseases but also to further explore the effects of smoking-related changes in DNA methylation on allergic diseases susceptibility.

**Fig. 1:**
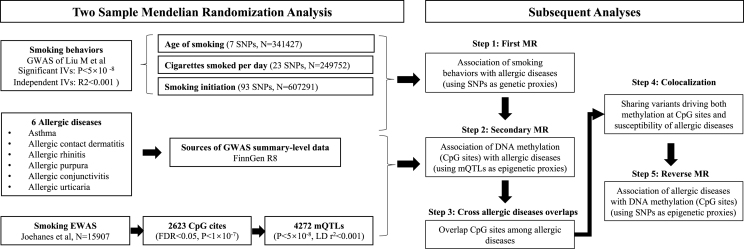
Study design.

In our study, we first conducted a two-sample MR analysis to investigate the relationships between smoking and genetic susceptibility to 6 allergic diseases. We selected 3 smoking behavior phenotypes (age of smoking, cigarettes smoked per day, smoking initiation) as specific measures of smoking. Subsequently, a second two-sample MR analysis was performed using mQTLs as IVs for CpG site methylation related to smoking in blood DNA, thereby revealing causal relationships at the epigenomic level. We focused on CpG sites that overlap across allergic diseases and explored their causal relationships with disease susceptibility. For significant overlapping CpG sites, colocalization analysis was conducted to access the impact of shared variants on both DNA methylation and disease susceptibility. Additionally, reverse MR analysis was performed to evaluate the reverse causal relationships between these CpG sites and genetic susceptibility to allergic diseases (Fig. 1).

**Fig. 2:**
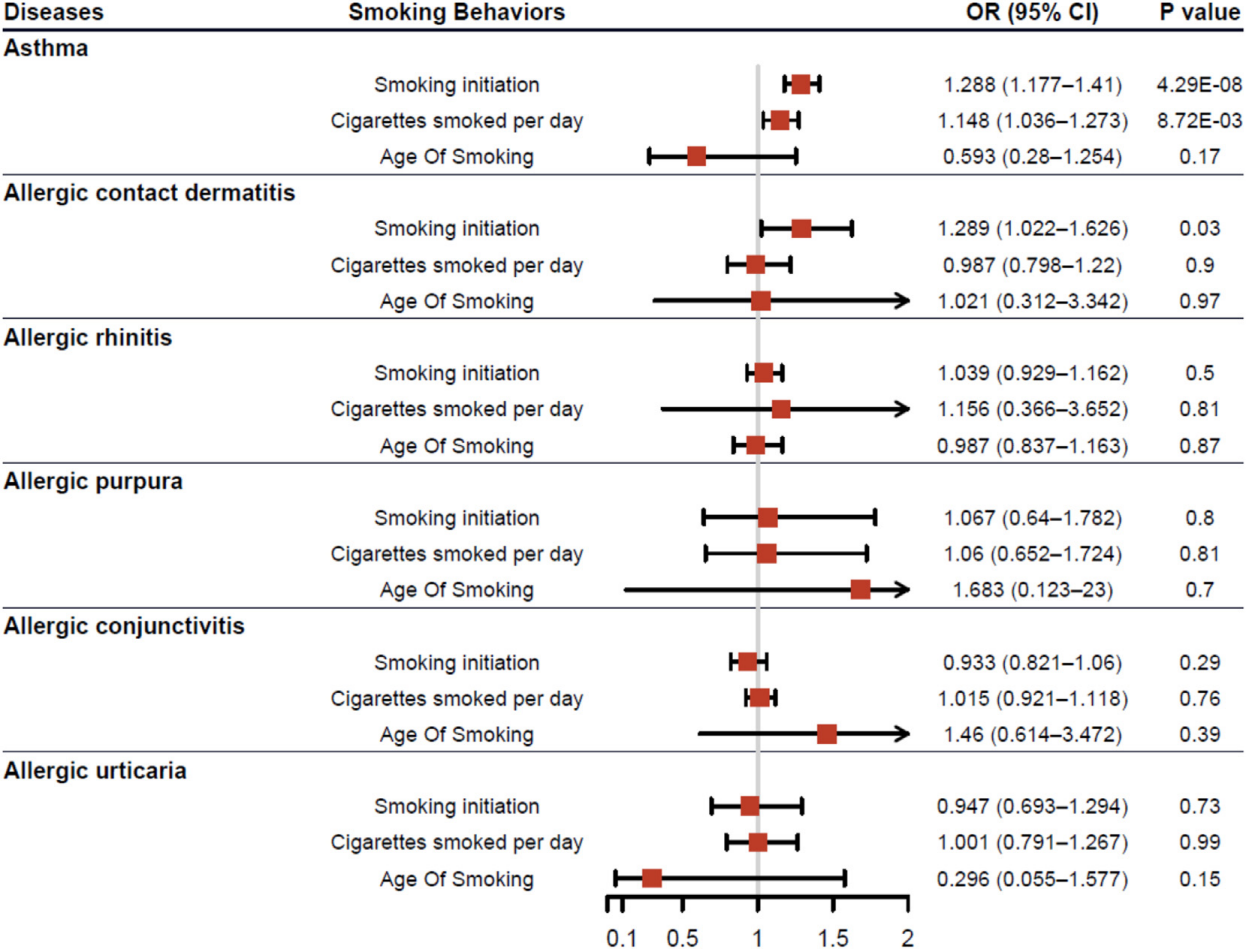
Two-sample Mendelian randomization estimates of smoking behaviours on allergic diseases risk. OR: odds ratios; CI: conﬁdence interval.

### Genetic instrumental variables of smoking behaviours

The IVs for 3 smoking behaviors were extracted from a Genome-Wide Association Study (GWAS) involving 1,232,091 participants of European descent.^25^ Initially, single-nucleotide polymorphisms (SNPs) were identified based on their significant associations (P value < 5 × 10⁻⁸) with specific smoking behaviors: age of smoking initiation (7 variants, N = 341,427), cigarettes smoked per day (23 variants, N = 249,752), and smoking initiation (93 variants, N = 607,291). These SNPs were then utilized as genetic instruments due to their robust and significant associations with the respective smoking behaviors. Linkage disequilibrium (LD) was computed based on the 1000 Genomes European reference panel. Genetic variants were carefully selected to ensure without LD, adhering to stringent criteria: an r^2^ < 0.001 and a clumping window exceeding 10,000 kb (Table S1).

### Epigenome-wide data of smoking-related DNA methylation

We utilized data from a genome-wide meta-analysis of 15,907 blood samples across 16 cohort studies within the Cohorts for Heart and Aging Research in Genetic Epidemiology Consortium.^26^ This analysis identified 2623 CpG sites with significantly different methylation levels between current smokers and never smokers (FDR < 0.05, P value < 1 × 10⁻⁷). To establish genetic proxies for these methylation changes, we accessed CpG-associated mQTLs from the Genetics of DNA Methylation Consortium (GoDMC), which includes over 30,000 participants. Methylation quantification was performed using Illumina Infinium HumanMethylation450 BeadChip on bisulfite-converted genomic DNA from blood samples.

### GWAS summary data of 6 allergic diseases

We sourced genetic data for 6 allergic diseases from the publicly available FinnGen biobank analysis (Round 8), with diagnoses based on ICD-10 codes. The European cohorts included data for 18,321 AC (ICD-10 code H10.1) cases and 324,178 controls, 3,846 ACD (ICD-10 code L20) cases and 306,909 controls, 780 AP (ICD-10 code D69.0) cases and 337,408 controls, 2,112 AU (ICD-10 code L50.0) cases and 331,270 controls, 9,707 AR (ICD-10 code J30) cases and 331,173 controls, and 37,253 cases of Asthma (ICD-10 code J45; J46) and 187,112 controls.

### Two-sample MR

We conducted 2 sequential two-sample MR analyses at the genomic and epigenomic levels. The initial MR analysis utilized SNPs associated with 3 smoking measurements as genetic proxies to assess their causal associations on multiple allergic diseases. Subsequently, we identified mQTLs as epigenetic proxies for CpG site methylation linked to smoking, aligning their effect alleles with smoking's impact on methylation. In addition, we conducted reverse MR analysis to explore a deeper understanding of the interaction between genetic susceptibility to allergic diseases and smoking related DNA methylation. For single-SNP exposures, we employed the Wald ratio for association estimation, while the inverse-variance weighted (IVW) method with random effects was used for combined exposure effects. Sensitivity analyses, including MR-Egger regression, Maximum Likelihood, and Simple Median, were applied to enhance robustness and address horizontal pleiotropy. Cochrane's Q value assessed the heterogeneity of genetic variants, and F-statistics evaluated the strength of instruments. Odds ratios and confidence intervals were calculated per standard deviation increase in genetically predicted smoking behavior and liability to allergic diseases. For overlapped CpG sites across multiple allergic diseases, we also conducted colocalization analysis. The threshold for statistical significance was determined by Bonferroni correction.

## Results

### Genomic MR results

Genetic variants for 3 smoking behaviors (smoking initiation, cigarettes smoked per day, and age of smoking) are listed in Table S2. The F-statistics for each IVs are all > 10, indicating no potential instrumental bias.

Based on the IVW method, the risk of asthma is significantly influenced by 2 smoking behaviors: smoking initiation (OR 1.288, 95% CI 1.177–1.410, P value = 4.29E-08) and cigarettes smoked per day (OR 1.148, 95% CI 1.036–1.273, P value = 8.72E-03). Additionally, a suggestive association (P value < 0.05) was observed between genetically predicted smoking initiation and ACD risk, with an OR of 1.289 (95% CI: 1.022–1.626). No associations were found between smoking behavior and risks of other allergic diseases, including AR, AP, AC, and AU (Fig. 2). Furthermore, MR-Egger analysis indicated no significant horizontal pleiotropy or outliers (Table S3).

### Epigenomic MR results

Following FDR correction, we identified smoking-associated DNA methylation correlations with asthma, using more than 1 mQTL as proxies, encompassing 46 CpG sites (Table 1). For the other five allergic diseases, we identified smoking-related DNA methylation correlations in 4 diseases—ACD, AR, AC, and AU—based on overlapping CpG sites (Table S4).

**Table 1:** Statistically significant CpGs summary in the secondary MR of smoking-related DNA methylation and allergic diseases

Diseases	With available mQTLs	P value < 0.05	FDR < 0.05
Asthma	1921	227	46
Allergic contact dermatitis	1921	92	3
Allergic rhinitis	1921	145	1
Allergic purpura	1921	95	0
Allergic conjunctivitis	1921	158	9
Allergic urticaria	1921	104	2

**Fig. 3:**
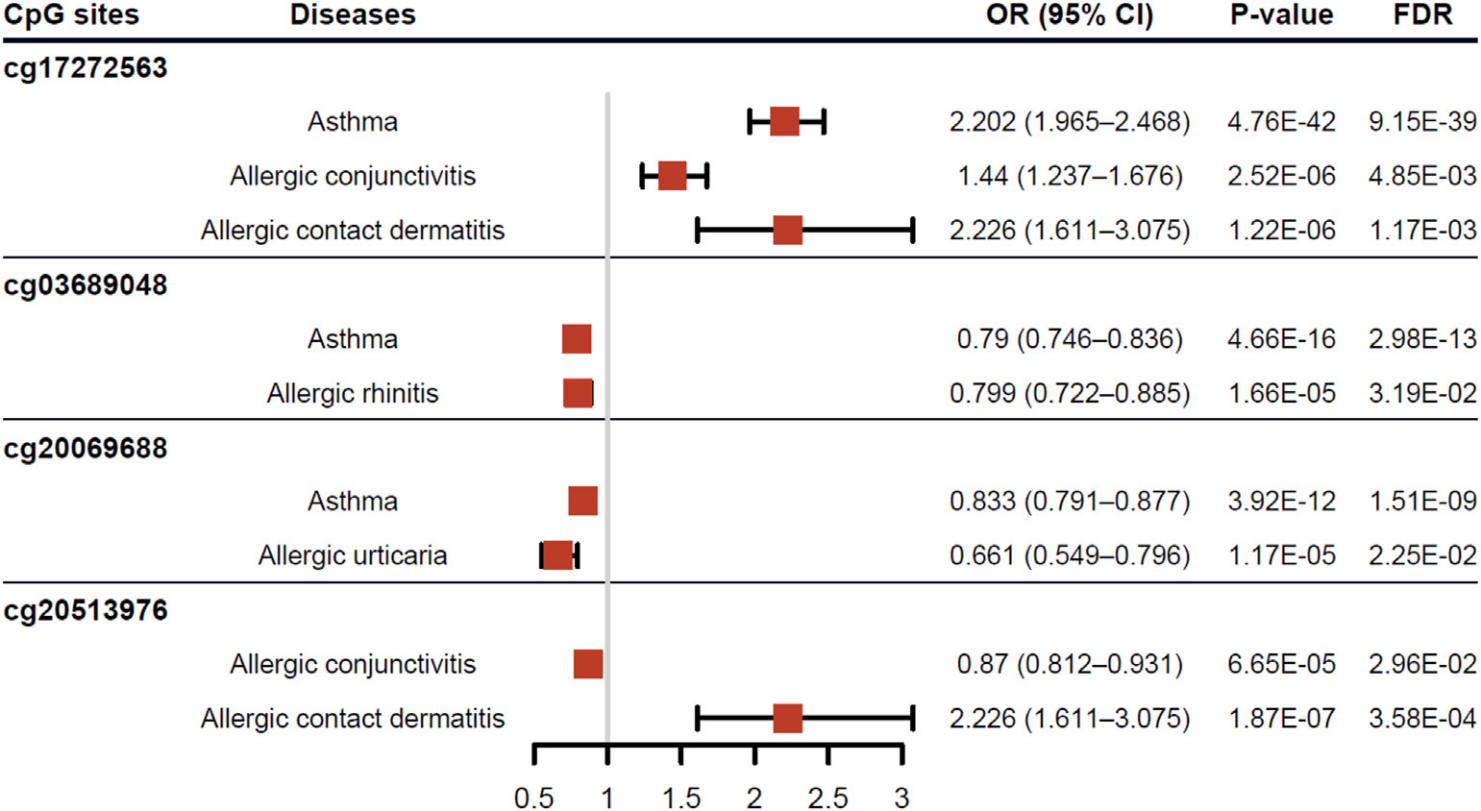
Evidence for the correlation between CpG Site methylation and allergic diseases susceptibility; OR: odds ratios; CI: conﬁdence interval.

A total of 55 CpG sites were found to significantly impact the risk of 5 site-specific allergic diseases, with 4 CpG sites exhibiting cross-allergic effects: cg17272563 (PRRT1), cg03689048 (BAT3), cg20069688 (STK19), and cg20513976 (LIME1) (Fig. 3). Most CpG sites impacted only 2 types of allergic diseases. For example, cg03689048 was associated with asthma (OR 0.79, 95% CI 0.746–0.836, P value = 4.66E-16) and AR (OR 0.799, 95% CI 0.722–0.885, P value = 1.66E-05); cg20069688 was associated with asthma (OR 0.833, 95% CI 0.791–0.877, P value = 3.92E-12) and AU (OR 0.661, 95% CI 0.549–0.796, P value = 1.17E-05); cg20513976 was associated with AC (OR 0.87, 95% CI 0.812–0.931, P value = 6.65E-05) and ACD (OR 2.226, 95% CI 1.611–3.075, P value = 1.87E-07). Notably, cg17272563 was associated with 3 allergic diseases: asthma (OR 2.202, 95% CI 1.965–2.468, P value = 4.76E-42), AC (OR 1.440, 95% CI 1.237–1.676, P value = 2.52E-6), and ACD (OR 2.226, 95% CI 1.611–3.075, P value = 1.22E-6) (Tables S4–S5). Additionally, colocalization analysis between these CpG sites and allergic diseases further confirmed these associations (PP.H3 + PP.H4 > 0.8) (Table S6).

### Reverse MR results

**Fig. 4:**
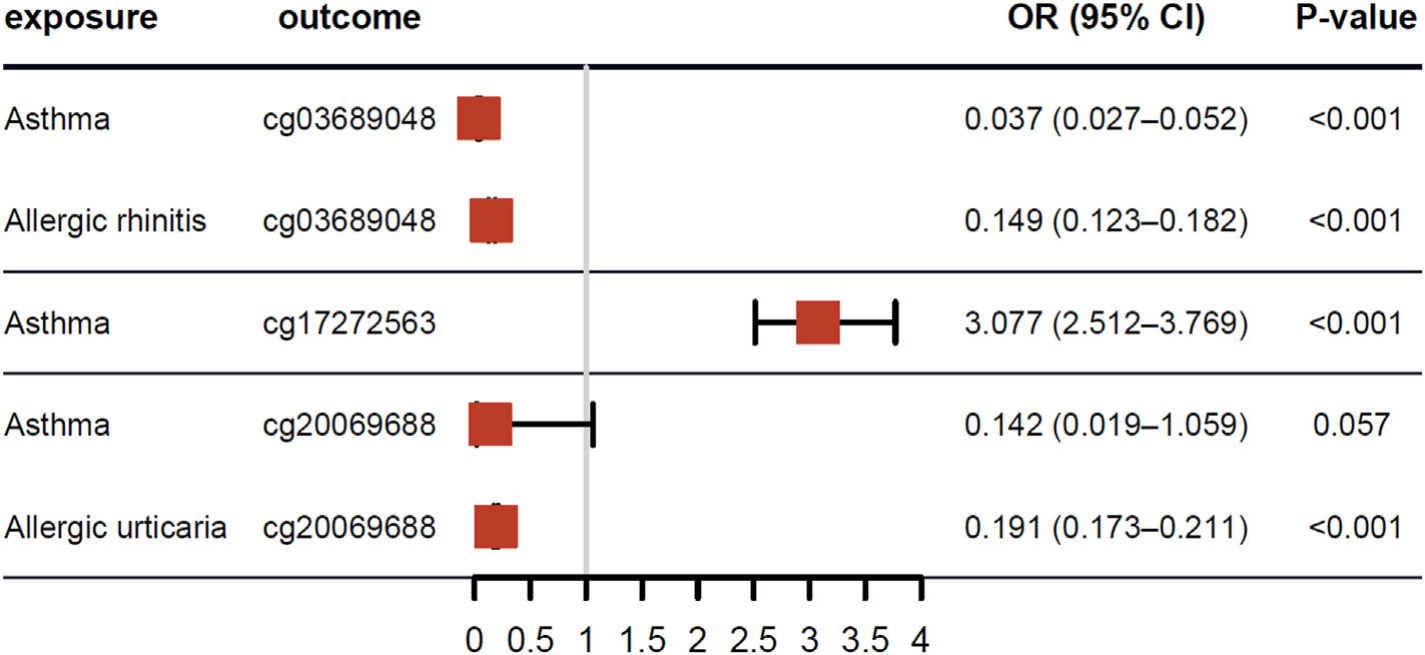
Association of allergic diseases with CpG Site methylation (using SNPs as epigenetic proxies). OR: odds ratios; CI: confidence interval.

Subsequently, we performed reverse MR analysis to investigate the causal relationships between the associations of allergic diseases and DNA methylation-associated overlapped CpG sites (using SNPs as epigenetic proxies). For the CpG sites in the secondary MR analysis, which showed significant causal effects on asthma and other allergic diseases, the reverse MR results confirmed consistent causal relationships with these allergic diseases. The findings aligned with the primary MR results (P value < 0.001) (Fig. 4). Complete reverse MR results are presented in Table S7.

## Discussion

In this study, we performed 2 separate two-sample MR analyses, both at the genomic and epigenomic levels, to explore the relationships between smoking and various allergic diseases. Additionally, we assessed the reverse causal relationships between smoking-related DNA methylation and allergic diseases at the epigenomic level. The first MR analysis demonstrated a strong causal relationship between smoking initiation, cigarettes smoked per day, and asthma risk. The second MR analysis highlighted the impact of DNA methylation at specific CpG sites in blood on multiple allergic diseases, with 4 CpG sites observed to have overlapping effects on these conditions. Reverse MR analysis further revealed bidirectional causal relationships between allergic diseases and DNA methylation at CpG sites cg03689048(BAT3), cg17272563 (PRRT1), and cg20069688 (STK19).

Our genomic MR analysis indicated that smoking behaviors have specific causal relationships with asthma among various allergic diseases. Numerous observational and experimental studies have explored the links between smoking and allergic diseases, examining how smoking behaviors may influence the onset and progression of these conditions. For instance, exposure to household smoking is closely associated with the development of food allergies in infancy and increased IgE production in later childhood. Moreover, infants exposed to smoking show an increased susceptibility to eczema and a heightened risk of developing allergic airway conditions during childhood.^27^ Certain components in cigarette smoke can impact the function of airway smooth muscles and the epithelial barrier, contributing to the development and progression of allergic airway diseases. These substances induce inflammation and oxidative stress, leading to alterations in mitochondrial structural proteins and disrupting mitochondrial function in airway smooth muscle, which are critical factors in respiratory diseases like asthma.^28^^,^^29^ The disruption of the epithelial barrier by these components increases the airway permeability to allergens and irritants, exacerbating allergic responses.^30^, ^31^, ^32^ A 2.5 years observational study reported similar findings, showing that current smokers and former smokers exhibited a significantly higher risk of developing asthma compared to never-smokers.^33^ This supports the causal relationship identified in our MR analysis between smoking behaviors and asthma. The elevated risk in both current and former smokers underscores the long-term impact of smoking on lung health and the development of respiratory diseases. Additionally, we found suggestive evidence indicating that smoking initiation may be linked to an increased risk of developing ACD. But we did not observe any significant impact of smoking behaviors on other allergic diseases such as AC, AU, AP, and AR. The occurrence of allergic diseases in different body sites leads to diverse clinical and pathological manifestations. Moreover, the pathogenesis of these diseases is complex, involving a multitude of factors including genetics, epigenetics, environmental influences, microbiota, and immune system functionality.^34^ Despite these complexities, previous studies have hinted at similar clues regarding contradictory findings. For instance, recent guidelines on AR highlight ongoing controversy and lack of consensus about smoking's impact on this condition.^35^ A previous study even posited that smoking might have a protective effect against AR.^36^ Further research is needed to fully understand the relationships between smoking and allergic diseases, particularly to unravel the complexities of immune system interactions with environmental factors like tobacco smoke.

Numerous studies have demonstrated the smoking impacts on the epigenetic landscape, particularly on DNA methylation across the entire genome.^15^, ^16^, ^17^ These methylation changes contribute to the increased risk of allergic diseases especially asthma.^18^, ^19^, ^20^, ^21^, ^22^ Our study identified that the CpG site cg17272563, located in the PRRT1 gene, exhibits a cross-disease effect, influencing the risk of developing asthma as well as AC and ACD. Reverse MR analysis also revealed a robust reverse causal relationship between cg17272563 (PRRT1) and asthma. This suggests that changes at this methylation site may influence asthma risk, and conversely, asthma may affect the methylation status of cg17272563 (PRRT1). PRRT1 is a component of native AMPA receptor (AMPAR) complexes in multiple brain regions. The expression of PRRT1 can influence AMPAR surface levels, phosphorylation status, and synaptic development and repair.^37^ AMPAR is tetrameric ligand-gated ion channels that mediate the transmission of glutamate signals in the brain. A recent study indicated that glutamate plays a role not only as a neurotransmitter but also as an immunomodulator.^38^ This dual function of glutamate adds a layer of complexity to our understanding of neural and immune system interactions. Furthermore, a study found that loratadine, an antihistamine commonly used to treat AR, can improve olfactory dysfunction in a rat model of AR and suppress the expression of AMPAR.^39^ A large-scale genome-wide interaction study also validated the association of PRRT1-related SNPs with the risk of lung cancer.^40^ The role of PRRT1 in allergic diseases may be attributed to the involvement of AMPAR in neuro-immune interactions, where the nervous and immune systems closely influence each other through signaling, especially during inflammatory responses. AMPAR mediates neurogenic inflammation and hyperalgesia by affecting Ca^2+^ permeability.^41^ In allergic diseases, particularly in allergic airway responses such as asthma and AR, allergen exposure often significantly activates afferent nerve terminals.^42^ AMPAR may contribute to this process by regulating airway hyperreactivity through its influence on signal transmission between neurons and bronchial smooth muscle, thereby enhancing airway sensitivity to allergens. Allergic airway inflammation can also induce the upregulation of AMPAR, potentially explaining our reverse MR results.^43^ Additionally, the activation of neurons may directly regulate allergic inflammation, which is typically driven by an excessive Th2-type immune response, involving the activation of inflammatory cells such as eosinophils and a large number of mast cells. Studies have shown that mast cells can form synapse-like connections with nerves in inflamed areas, and the infiltration of eosinophils in allergic inflammation is also nerve-related, leading to the release of allergic inflammatory mediators and thereby intensifying the inflammatory response—AMPAR may also be involved in this process.^44^ Our study contributes a novel epigenetic perspective, proposing that PRRT1 may elevate the asthma risk through DNA methylation modifications. Consequently, it is essential to further explore the mechanisms of interaction between PRRT1 and asthma. Additionally, the development of therapies targeting the PRRT1-related pathway holds significant potential.

This research has identified a significant association between cg03689048 (BAT3) and a reduced risk of asthma and AR. Smoking-related methylation changes at cg03689048 (BAT3) may affect the expression of BAT3, thus influencing the risk of developing the allergic airway diseases asthma and AR. BAT3 is encoded by the BAG6 gene, located in the MHC class III gene cluster on chromosome 6, which encodes genes that play critical roles in immune function.^45^ Study found that BAT3 can influence the activity of various immune cells, such as regulate the functional state of dendritic cells (DCs), which are crucial in shaping T cell responses, including T cell anergy, T cell deletion, or conversion to FOXP3^+^ regulatory T cells (Tregs).^46^ BAT3 regulates NK cell activity, influencing their antitumor function.^47^ Additionally, BAT3 promotes T cell responses and regulate macrophage function.^48^ In allergic diseases, BAT3 may relieve Th2-type immune responses by reducing immune cell recognition and presentation of environmental allergens. BAT3 also acts as a co-chaperone of heat shock protein 70 (HSP70) and is involved in various developmental processes, cellular stress responses, and cell viability.^49^ HSP70 participates in several allergy-related biological processes, and its upregulation is associated with asthma in children with AR.^50^ House dust mites (HDM) can induce epithelial inflammation by upregulating the TLR4-NF-κB pathway through HSP70.^51^ Previous study has suggested that HSP70 may exert protective effects in allergic diseases, such as downregulating eosinophilia, reducing Th2 cytokines (e.g., IL-4, IL-5, IL-13), and lowering allergen-specific IgE levels.^52^ Overall, the development of allergic diseases is often accompanied by inflammatory damage to cells and tissues, and BAT3 may assist cells in responding to stress and modulating immune responses through its interaction with HSP70. BAT3 may thereby influence the immune response to allergens and regulate the extent of local inflammation. These findings may also corroborate our results, which show a significant association between BAT3 and a reduced risk of asthma and AR. Smoking may induce changes at the DNA methylation site cg03689048 (BAT3), potentially leading to immune function decline. These hypotheses require further experimental validation to confirm the causative links between smoking, DNA methylation changes at cg03689048 (BAT3), and potential immune function decline. Future research should include observational and functional studies to unravel the precise biological mechanisms by which smoking-induced epigenetic modifications at this site may influence BAT3 expression and its subsequent impact on the immune system.

Our epigenomic MR analysis also indicates that cg20069688 (STK19) has a causal influence on asthma and AU through methylation modification. STK19 is a nuclear kinase that phosphorylates RNA-binding proteins during transcription and is involved in DNA repair and nuclear signal transduction during active transcription.^53^ STK19, as a novel NRAS activator, is considered to play a tumorigenic activating role in melanocytes.^54^ Kinases play a crucial role in allergic inflammation, acting as key regulators in the signaling pathways that mediate immune responses. They are significantly involved in allergic diseases such as asthma and anaphylaxis by regulating mast cell activation and cytokine release.^55^ As a member of the kinase family, SKT19 may also regulate immune responses in allergic diseases. However, given the limited evidence observed regarding STK19 and allergic diseases, further empirical validation based on genetics or population studies is necessary to substantiate the association between the STK19 gene and allergic diseases.

In this study, we comprehensively examined the relationship between smoking behaviors and 6 allergic diseases to elucidate the differing risks of allergic conditions associated with smoking and its sensitizing effects on the body. We used genetic instruments sourced from the most contemporary GWAS database to enhance the rigor of our findings. MR analysis was employed to minimize the impact of confounding variables. By utilizing both SNPs and mQTLs as IVs, we provided corroborative evidence from genetic and epigenetic perspectives, highlighting the role of methylation modifications in the etiology of allergic diseases.

The MR method, while powerful, has inherent limitations that require careful consideration. A key limitation is the potential violation of MR assumptions: relevance, independence, and exclusion restriction. We addressed the relevance assumption by selecting genetic variants significantly associated with smoking behaviors and CpG methylation levels from large, well-powered GWAS datasets. We mitigated the independence assumption by employing a two-sample MR design with independent datasets from the FinnGen Biobank, which reduces the likelihood of shared confounders. Despite these measures, unmeasured pleiotropy—where genetic variants affect the outcome through other pathways—remains a challenge. We conducted sensitivity analyses to account for horizontal pleiotropy; however, it remained a significant concern, particularly when phenotypes were inferred from a limited set of SNPs. The exclusion restriction assumption is difficult to fully validate, particularly for complex traits such as smoking and DNA methylation. We addressed this through reverse MR and colocalization analyses; however, tissue-specific methylation differences, lack of temporal data, and European ancestry bias in GWAS cohorts limit the generalizability of our findings. Additionally, due to the lack of individualized clinical data in MR frameworks, we were unable to develop an Integrated Prognostic Assessment (IPA) index, which could provide a more nuanced understanding of individual risks.

Our findings have important implications for clinical practice and public health. Given the identified causal relationships between smoking behaviors and increased risk of allergic diseases, our results highlight the importance of preventive strategies against smoking to reduce the risk of conditions like asthma and AR. Health education programs could use these findings to emphasize the added risk smoking poses to respiratory health, especially in populations predisposed to allergic diseases. Clinicians managing allergic patients should incorporate smoking cessation into treatment plans to potentially improve outcomes by reducing inflammation and modifying epigenetic risk factors. Personalized smoking cessation programs could be particularly beneficial for individuals with high genetic susceptibility to smoking-related allergic diseases. While our current data limitations precluded the calculation of an IPA index, we recognize that such an index could strengthen the clinical utility of our findings. Personalized smoking cessation programs could be particularly beneficial for individuals with high genetic susceptibility to smoking-related allergic diseases, especially if combined with detailed prognostic assessments like the IPA index in future studies.

Future research should focus on validating these findings in more diverse populations to enhance generalizability. Additionally, studies are needed to investigate tissue-specific methylation patterns and the temporal dynamics of methylation changes related to smoking exposure. Understanding the biological mechanisms underlying the identified CpG sites and their impact on allergic diseases development, along with longitudinal data, would clarify causal pathways and guide targeted interventions. Moreover, collaboration with cohorts possessing clinical and phenotypic data could facilitate comprehensive prognostic assessments. Incorporating the IPA index into such studies would enable a deeper understanding of individual risk profiles, leading to more personalized and effective strategies for preventing smoking-related allergic diseases.

## Conclusion

In summary, our study has established a genetic connection between smoking behaviors and various allergic diseases, notably asthma, providing novel epigenetic insights. Specifically, smoking-induced DNA methylation changes at certain CpG sites are implicated in the pathogenesis of these conditions. For instance, cg17272563 (PRRT1) not only showed a significant association with asthma but also satisfied reverse MR analysis, highlighting its role in the causal pathway of asthma development. Other notable CpG sites, such as cg03689048 (BAT3) and cg20069688 (STK19), demonstrated cross-allergic influences, suggesting their broad impact on allergic diseases.

## Author contributions

Conception: Junhao Tu. Interpretation or analysis of data: Junhao Tu, Wei Wan, Binxiang Tang, Fan jiang, Qing Luo, and Jinyang Wen. Preparation of the manuscript: Junhao Tu. Revision for important intellectual content: Junhao Tu. Supervision: Jing Ye.

## Consent for publication

All authors have seen and approved the manuscript and agreed to the publication of the work.

## Ethics approval and consent to participate

This article incorporates human participants' data obtained from earlier studies. In each corresponding original research, all participants provided their informed consent. The foundation of our study lies in the analysis of large-scale GWAS datasets, rather than individual-level data. Consequently, there was no requirement for ethical approval in this context.

## Funding

This study was supported by the 10.13039/501100001809National Natural Science Foundation of China (grant nos. 81860182 and 82360219), Jiangxi Nutrition and Health Management Medical Research Institute Cultivation Project (2022-PYXM-05) and Central Funds Guiding the Local Science and Technology Development (20221ZDG020066).

## Data availability

Data contains in the manuscript are provided within the article. Public data can be found here: (https://www.finngen.fi/en/access_results).

## Declaration of competing interest

All authors declare no conflict of interest.

## References

[1] Wesemann D.R., Nagler C.R. (2016). The microbiome, timing, and barrier function in the context of allergic disease. Immunity.

[2] Zheng T., Yu J., Oh M.H., Zhu Z. (2011). The atopic march: progression from atopic dermatitis to allergic rhinitis and asthma. Allergy Asthma Immunol Res.

[3] Gibson A., Deshpande P., Campbell C.N. (2023). Updates on the immunopathology and genomics of severe cutaneous adverse drug reactions. J Allergy Clin Immunol.

[4] Gabryszewski S.J., Hill D.A. (2021). One march, many paths: insights into allergic march trajectories. Ann Allergy Asthma Immunol.

[5] Gómez R.M., Croce V.H., Zernotti M.E., Muiño J.C. (2021). Active smoking effect in allergic rhinitis. World Allergy Organ J.

[6] Salehi M., Bakhshaee M., Ashtiani S.J., Najafi M., Sehatbakhsh S., Hossainzadeh M. (2014). Parental smoking and allergic rhinitis in children. Int Forum Allergy Rhinol.

[7] Kantor R., Kim A., Thyssen J.P., Silverberg J.I. (2016). Association of atopic dermatitis with smoking: a systematic review and meta-analysis. J Am Acad Dermatol.

[8] Li S., Wei J., Hu Y. (2023). Long-term effect of intermediate particulate matter (PM(1)(-)(2.5)) on incident asthma among middle-aged and elderly adults: a national population-based longitudinal study. Sci Total Environ.

[9] Shinohara M., Matsumoto K. (2017). Fetal tobacco smoke exposure in the third trimester of pregnancy is associated with atopic eczema/dermatitis syndrome in infancy. Pediatr Allergy Immunol Pulmonol.

[10] Hingorani A., Humphries S. (2005). Nature's randomised trials. Lancet.

[11] Chen Y., Xu X., Wang L. (2022). Genetic insights into therapeutic targets for aortic aneurysms: a Mendelian randomization study. EBioMedicine.

[12] Chen Y., Sun Y., Wang L., Xu K., Wang D.W. (2023). Genetic insights into associations of multisite chronic pain with common diseases and biomarkers using data from the UK Biobank. Br J Anaesth.

[13] Tu J., Wen J., Luo Q., Li X., Wang D., Ye J. (2024). Causal relationships of metabolites with allergic diseases: a trans-ethnic Mendelian randomization study. Respir Res.

[14] Skaaby T., Taylor AE., Jacobsen RK. (2017). Investigating the causal effect of smoking on hay fever and asthma: a Mendelian randomization meta-analysis in the CARTA consortium. Sci Rep.

[15] Peng G., Xi Y., Bellini C. (2022). Nicotine dose-dependent epigenomic-wide DNA methylation changes in the mice with long-term electronic cigarette exposure. Am J Cancer Res.

[16] Heikkinen A., Bollepalli S., Ollikainen M. (2022). The potential of DNA methylation as a biomarker for obesity and smoking. J Intern Med.

[17] Laqqan M.M., Yassin M.M. (2022). Cigarette heavy smoking alters DNA methylation patterns and gene transcription levels in humans spermatozoa. Environ Sci Pollut Res Int.

[18] Herrera-Luis E., Rosa-Baez C., Huntsman S. (2023). Novel insights into the whole blood DNA methylome of asthma in ethnically diverse children and youth. Eur Respir J.

[19] Koo H.K., Morrow J., Kachroo P. (2021). Sex-specific associations with DNA methylation in lung tissue demonstrate smoking interactions. Epigenetics.

[20] van Breugel M., Qi C., Xu Z. (2022). Nasal DNA methylation at three CpG sites predicts childhood allergic disease. Nat Commun.

[21] Xu C.J., Söderhäll C., Bustamante M. (2018). DNA methylation in childhood asthma: an epigenome-wide meta-analysis. Lancet Respir Med.

[22] Gibson F., Hanly A., Grbic N. (2022). Epigenetic dysregulation in autoimmune and inflammatory skin diseases. Clin Rev Allergy Immunol.

[23] Bellanti J.A. (2019). Genetics/epigenetics/allergy: the gun is loaded … but what pulls the trigger?. Allergy Asthma Proc.

[24] Luo Y., Zhou B., Zhao M., Tang J., Lu Q. (2014). Promoter demethylation contributes to TSLP overexpression in skin lesions of patients with atopic dermatitis. Clin Exp Dermatol.

[25] Liu M., Jiang Y., Wedow R. (2019). Association studies of up to 1.2 million individuals yield new insights into the genetic etiology of tobacco and alcohol use. Nat Genet.

[26] Joehanes R., Just AC., Marioni RE. (2016). Epigenetic signatures of cigarette smoking. Circ Cardiovasc Genet.

[27] Wang Y.W., Yeh KW., Huang JL. (2023). Longitudinal analysis of the impact of smoking exposure on atopic indices and allergies in early childhood. World Allergy Organ J.

[28] Borkar N.A., Thompson MA., Bartman CM. (2023). Nicotine affects mitochondrial structure and function in human airway smooth muscle cells. Am J Physiol Lung Cell Mol Physiol.

[29] Ferraro M., Di Vincenzo S., Lazzara V. (2023). Formoterol exerts anti-cancer effects modulating oxidative stress and epithelial-mesenchymal transition processes in cigarette smoke extract exposed lung adenocarcinoma cells. Int J Mol Sci.

[30] Ghosh B., Chengala PP., Shah S. (2023). Cigarette smoke-induced injury induces distinct sex-specific transcriptional signatures in mice tracheal epithelial cells. Am J Physiol Lung Cell Mol Physiol.

[31] Chen R., Hui KP., Liang Y. (2023). SARS-CoV-2 infection aggravates cigarette smoke-exposed cell damage in primary human airway epithelia. Virol J.

[32] Ham J., Kim J., Sohn KH. (2022). Cigarette smoke aggravates asthma by inducing memory-like type 3 innate lymphoid cells. Nat Commun.

[33] Piipari R., Jaakkola J.J., Jaakkola N., Jaakkola M.S. (2004). Smoking and asthma in adults. Eur Respir J.

[34] Wang J., Zhou Y., Zhang H. (2023). Pathogenesis of allergic diseases and implications for therapeutic interventions. Signal Transduct Targeted Ther.

[35] Wise S.K., Lin SY., Toskala E. (2018). International consensus statement on allergy and rhinology: allergic rhinitis. Int Forum Allergy Rhinol.

[36] Wang S., Qi L., Wei H., Jiang F., Yan A. (2022). Smoking behavior might affect allergic rhinitis and vasomotor rhinitis differently: a mendelian randomization appraisal. World Allergy Organ J.

[37] Matt L., Kirk LM., Chenaux G. (2018). SynDIG4/Prrt1 is required for excitatory synapse development and plasticity underlying cognitive function. Cell Rep.

[38] Pacheco R., Oliva H., Martinez-Navío JM. (2006). Glutamate released by dendritic cells as a novel modulator of T cell activation. J Immunol.

[39] Li S., Zhang X., Li Z. (2020). Desloratadine ameliorates olfactory disorder and suppresses AMPA receptor GluA1 expression in allergic rhinitis rat. Arch Immunol Ther Exp.

[40] Zhang R., Shen S., Wei Y. (2022). A large-scale genome-wide gene-gene interaction study of lung cancer susceptibility in Europeans with a trans-ethnic validation in asians. J Thorac Oncol.

[41] Kopach O., Dobropolska Y., Belan P., Voitenko N. (2023). Ca(2+)-Permeable AMPA receptors contribute to changed dorsal horn neuronal firing and inflammatory pain. Int J Mol Sci.

[42] Omori Y., Andoh T., Shirakawa H., Ishida H., Hachiga T., Kuraishi Y. (2009). Itch-related responses of dorsal horn neurons to cutaneous allergic stimulation in mice. Neuroreport.

[43] Saitoh B.Y., Tanaka E., Yamamoto N. (2021). Early postnatal allergic airway inflammation induces dystrophic microglia leading to excitatory postsynaptic surplus and autism-like behavior. Brain Behav Immun.

[44] Undem B.J., Taylor-Clark T. (2014). Mechanisms underlying the neuronal-based symptoms of allergy. J Allergy Clin Immunol.

[45] Spies T., Blanck G., Bresnahan M., Sands J., Strominger J.L. (1989). A new cluster of genes within the human major histocompatibility complex. Science.

[46] Iberg C.A., Jones A., Hawiger D. (2017). Dendritic cells as inducers of peripheral tolerance. Trends Immunol.

[47] Rangachari M., Zhu C., Sakuishi K. (2012). Bat3 promotes T cell responses and autoimmunity by repressing Tim-3–mediated cell death and exhaustion. Nat Med.

[48] Grover A., Izzo A.A. (2012). BAT3 regulates Mycobacterium tuberculosis protein ESAT-6-mediated apoptosis of macrophages. PLoS One.

[49] Corduan A., Lecomte S., Martin C., Michel D., Desmots F. (2009). Sequential interplay between BAG6 and HSP70 upon heat shock. Cell Mol Life Sci.

[50] Fagotti A., Lucentini L., Simoncelli F. (2022). HSP70 upregulation in nasal mucosa of symptomatic children with allergic rhinitis and potential risk of asthma development. Sci Rep.

[51] Liu C., Huang XL., Liang JP. (2021). Serum-derived exosomes from house dust mite-sensitized Guinea pigs contribute to inflammation in BEAS-2B cells via the TLR4-NF-κB pathway. Mol Med Rep.

[52] Shevchenko M., Servuli E., Albakova Z., Kanevskiy L., Sapozhnikov A. (2020). The role of heat shock protein 70 kDa in asthma. J Asthma Allergy.

[53] Boeing S., Williamson L., Encheva V. (2016). Multiomic analysis of the UV-induced DNA damage response. Cell Rep.

[54] Yin C., Zhu B., Zhang T. (2019). Pharmacological targeting of STK19 inhibits oncogenic NRAS-driven melanomagenesis. Cell.

[55] Crozier R.W.E., Fajardo V.A., MacNeil A.J. (2023). Targeting glycogen synthase kinase 3 with CHIR99021 negatively regulates allergen-induced mast cell activation. Eur J Immunol.

